# Experimental Results of Underwater Cooperative Source Localization Using a Single Acoustic Vector Sensor

**DOI:** 10.3390/s130708856

**Published:** 2013-07-12

**Authors:** Paulo Felisberto, Orlando Rodriguez, Paulo Santos, Emanuel Ey, Sérgio M. Jesus

**Affiliations:** Laboratory of Robotics and Systems in Engineering and Science (LARSyS), University of Algarve, Campus de Gambelas, 8005-139 Faro, Portugal; E-Mails: orodrig@ualg.pt (O.R.); pjsantos@ualg.pt (P.S.); emanuel.ey@gmail.com (E.E.); sjesus@ualg.pt (S.M.J.)

**Keywords:** vector sensors, source localization, ray backpropagation

## Abstract

This paper aims at estimating the azimuth, range and depth of a cooperative broadband acoustic source with a single vector sensor in a multipath underwater environment, where the received signal is assumed to be a linear combination of echoes of the source emitted waveform. A vector sensor is a device that measures the scalar acoustic pressure field and the vectorial acoustic particle velocity field at a single location in space. The amplitudes of the echoes in the vector sensor components allow one to determine their azimuth and elevation. Assuming that the environmental conditions of the channel are known, source range and depth are obtained from the estimates of elevation and relative time delays of the different echoes using a ray-based backpropagation algorithm. The proposed method is tested using simulated data and is further applied to experimental data from the Makai'05 experiment, where 8–14 kHz chirp signals were acquired by a vector sensor array. It is shown that for short ranges, the position of the source is estimated in agreement with the geometry of the experiment. The method is low computational demanding, thus well-suited to be used in mobile and light platforms, where space and power requirements are limited.

## 1. Introduction

This paper proposes a single sensor based three-dimensional localization method that, by taking advantage of the spatial filtering capabilities of a vector sensor, allows for a low computational demanding implementation, suitable for light real-time systems. An acoustic vector sensor (VS) is a device that measures the three orthogonal components of the particle velocity simultaneously with the pressure field at a single position in space. Vector sensors have been long used for target localization by the US Navy, due to their inherent spatial filtering capabilities [1]. In the early 1990s, in a paper that received considerable attention, D'Spain *et al.* [2] presented results for single-element and full array beamformed data acquired by an array of 16 vector sensors, the directional frequency analysis and recording (DIFAR) array. During the last two decades, several authors have conducted research on the signal processing theory of vector sensors (see, for instance, [3–5] and the references therein). Although the majority of that work is related to direction of arrival estimation, in the last decade, vector sensors have been proposed in other fields, like port and waterway security [6], underwater communications [7], geoacoustic inversion [8–10] and geophysics [11].

Taking advantage of the intrinsic spatial filtering capability of a vector sensor (a typical VS presents a figure of height directivity pattern and a directivity index of 4.8 dB [12]), the usage of a single vector sensor for the direction of arrival estimation (azimuth and elevation) was considered by several theoretical and simulation studies. Due to the collocation of the pressure and the orthogonal particle velocity sensing elements in a single vector sensor device, the direction of arrival algorithms can be frequency invariant, thus computationally simple direction of arrival (DOA) algorithms can be used for *a priori* unknown and time-varying broadband signals in the presence of spatially distributed broadband interferences [13]. Azimuth and elevation algorithms for tracking of a passive source using a single vector sensor were proposed by Liu *et al.* [14] based in Kalman filters and Awad and Wong [15] based in a recursive least-squares. The performance of both methods were compared in [15] considering a simulation scenario.

Due to multipath, in shallow water environments, the waveform impinging on a receiver is a sum of different echoes. Rahamim *et al.* [16] proposed various vector sensor array (VSA)-based direction of arrival estimators for multipath environments and evaluated their performance using simulations. Arunkumar and Anand [17] proposed a method for three-dimensional (3D) source localization of a narrowband source using a vector sensor array. Their method is based in a normal mode representation of a range-independent shallow ocean. It is shown that the azimuth of the source can be estimated directly from the horizontal components measured at a vector sensor array, the range is obtained by closed form, and the depth is estimated by a matched-field approach. Hurtado and Nehorai [18] analyzed the performance of a passive direction of arrival and a range estimation method of a source in the air above the ocean based on the interference between the direct and sea-surface reflected field impinging on polarization-sensitive (electromagnetic) sensors.

Thanks to technological advances and small size, low noise underwater acoustic vector sensors with improved dynamic range and bandwidth are becoming available [19]. Those compact sensors are well-suited to be used in light systems, where space and computational resources are limited and energy consumption is of concern, as, for example, in autonomous underwater vehicles (AUV) and similar mobile platforms. Hawkes and Nehorai [20] proposed a fast broadband intensity-based algorithm for determining three-dimensional localization of a source using distributed vector sensors situated on a reflecting boundary. The method considers that each vector sensor is impinged on by a direct echo, which determines the elevation of the source, and by a reflected echo. The reflected echo does not affect the azimuth estimate, as long as the environment is considered homogeneous, but it introduces errors in elevation estimation. The authors proposed a method that filters out the reflected echo, thus achieving a more accurate elevation estimate of the source. The performance of the method was shown for a simulated environment, where the three-dimensional localization of the source was obtained from a set of azimuth and elevation estimates obtained from distributed vector sensors.

The present paper shows that the azimuth, range and depth of a high frequency broadband cooperative source, slowly moving (<0.3 m/s) in a shallow water environment, can be tracked in the presence of multipath using a single vector sensor. The azimuth and elevation of the echoes impinging on the vector sensor are estimated from the amplitude of the particle velocity components using a least squares-based algorithm. Then, a ray backpropagation method [21] is applied to estimate source range and depth, where ray trajectories are launched from the receiver at the elevation angles estimated from the various echoes. Afterwards, the range and depth estimates are obtained by least squares minimization of an objective function that combines the ray trajectories and the relative travel times estimated in the previous stage. The range and depth estimation method can be implemented with a single forward ray tracing model run. Additionally, when only the direct and the surface-reflected echoes are considered, source range and depth can be estimated using the source image method. Although the method requires *a priori* a complete record of the source signal, it is very simple to implement even in light platforms, thus suitable for real-time localization and tracking of cooperative sources. The proposed method is tested with simulated data and applied to a data set acquired during the Makai Experiment (Makai'05) held in September 2005, off the coast of Kauai Island (Hawaii, HI, USA) using a Wilcoxon TV-001 vector sensor device [22]. The orientation of the *x*- and *y*-axes of the vector sensor, initially unknown, is determined from the ship self noise using an intensity method [23], based on the inner product between the sample pressure and the various particle velocity components. The results obtained from a 8–14 kHz chirp signal transmitted from a cooperative source are in agreement with the known geometry of the experiment, showing that the 3D localization of the source is achieved for ranges until 500 m (the azimuth alone was tracked along a 2 km transect [24]). The method presented herein is very fast when compared with single hydrophone methods [25–28], which require a large number of time-consuming forward model runs associated with complex optimization procedures.

This paper is organized as follows: Section 2 presents the theoretical framework considered in the data processing and analysis. In Section 3, the proposed method is tested in a simulated scenario. Section 4 shows and discusses the results obtained on a real data set, and Section 5 summarizes the paper. Preliminary results of this work were presented in [29].

## 2. Theoretical Framework

### 2.1. The Measurement Model

In the following, a vector sensor is considered that measures the pressure, *p*(*t*), and the three orthogonal components of the particle velocity, *v_x_*(*t*), *v_y_*(*t*) and *v_z_*(*t*), along the *x*-, *y*- and *z*-axes, respectively. The vector sensor is located at the origin of the Cartesian coordinate system, *x*–*y* being the horizontal plane and *x*–*z* the vertical plane. The azimuth, Θ (−180° ≤ Θ ≤ 180°), and elevation, Φ (−90° ≤ Φ ≤ 90°), are defined in a conventional manner.

Without loss of generality, it is assumed that the signal impinging on the vector sensor is in the far-field and band-limited; thus, pressure, *p*(*t*), is related to particle velocity, v, by:

(1) $$\frac{\partial \text{v}}{\partial t}=-\frac{1}{\rho_{0}}\nabla p$$

where *ρ*_0_ is the medium density and ∇ is the gradient operator.

Assuming a monochromatic signal of frequency, *ω*, one can write that:

(2) $$\text{v}=-\frac{1}{j\omega \rho_{0}}\nabla p$$

where *j* is the imaginary unit. In the far-field of a free-space environment with sound speed, *c*_0_, the wavefront is planar; thus:

(3) $$\text{v}=\frac{1}{\rho_{0}c_{0}}p\text{u}$$

where **u** = [*u_x_*, *u_y_*, *u_z_*] is the unit vector pointing to the source (thus, in the opposite direction of the wavefront propagation).

When a field due to a point source in the far-field is sampled by a vector sensor with small dimension compared to the signal wavelength, then the wavefront can by considered planar. Thus, from Equation (3), the measured pressure and particle velocity components are related by:

(4) $$\begin{array}{ccccc} \left[\begin{array}{c} p(t)\\ v_{x}(t)\\ v_{y}(t)\\ v_{z}(t) \end{array}\right] & = & \left[\begin{array}{c} s(t)\\ \alpha s(t)u_{x}\\ \alpha s(t)u_{y}\\ \alpha s(t)u_{z} \end{array}\right] & + & \left[\begin{array}{c} n(t)\\ n_{x}(t)\\ n_{y}(t)\\ n_{z}(t) \end{array}\right] \end{array}$$

where *s*(*t*) is the source waveform, *u_x_* = cos(Φ*_s_*) cos(Θ*_s_*), *u_y_* = cos(Φ*_s_*) sin(Θ*_s_*), *u_z_* = sin(Φ*_s_*), Φ*_s_* is the source elevation and Θ*_s_* is the source azimuth. The proportionality factor, *α*, arises directly from Equation (3), but in a more general setting, it can also account for any existing proportionality in the output streams of a vector sensor device, due to the various electro-mechanical principles used to measure pressure and particle velocity. In Equation (4), *n*(*t*) represents additive pressure noise, and *n_x_*(*t*), *n_y_*(*t*), *n_z_*(*t*) its particle velocity counterparts. A common assumption is that signal and noise are uncorrelated both in time and space. The cross-correlation between the four vector sensor components have been studied by several authors [30,31]. It was demonstrated that in the presence of azimuthally isotropic noise, the horizontal particle velocity and the pressure components are mutually uncorrelated. Moreover, if the noise is spherically symmetric, the vertical particle velocity term is also uncorrelated with the other noise terms. Furthermore, the noise power at the pressure channel is equal to the sum of noise power at the so-called pressure equivalent of particle velocity [31], which is obtained by the product of the particle velocity by −*ρ*_0_*c* [23].

### 2.2. Intensity-Based Azimuth Estimation

Intensity-based source direction estimation was considered in the pioneering work of D'Spain *et al.* [2]. Later on, Nehorai and Paldi [23] revisited the method and analyzed its statistical performance in terms of the Cramér-Rao bound and mean square angular error. The method is based on the cross-correlation between the pressure measurements and the various components of the particle velocity, which allow one to estimate the factors, *αu_x_*, *αu_y_* and *αu_z_*, and, subsequently, the direction of the impinging wavefront. Taking into account that the signal and the noise are zero mean uncorrelated processes and the pressure and the *x* component of the particle velocity in Equation (4), one can write the cross-correlation at lag 0 between these two vector sensor components as:

(5) $$E[v_{x}(t)p(t)]=\alpha u_{x}E\;[s^{2}(t)]+E\;[n_{x}(t)n(t)]$$

where *E* [ ] is the expectation operator and *E* [*s*^2^(*t*)] represents the energy of the signal as seen by the vector sensor. The term, *E* [*n_x_*(*t*)*n*(*t*)], represents the cross-correlation (at lag 0) between the pressure and the *x* component of the particle velocity noise. For a number of cases, in the presence of isotropic noise, this term can be assumed to be zero (see [30,31]) or, in practice, a small fraction of the signal power term (high signal to noise ratios (SNR) situation). Thus, for high SNR, *αu_x_* can be estimated directly from the cross-correlation (at lag 0) between the pressure and the *x* component of particle velocity. Similar analysis holds for the cross-correlation between the pressure and the *y* component of the particle velocity.

Assuming that *s*(*t*) and the noise components are ergodic processes, a possible estimator for the azimuthal direction of the source signal, Θ*_s_*, is given by:

(6) $${\hat{\Theta }}_{s}=\arctan\frac{\langle v_{y}(t)p(t)\rangle }{\langle v_{x}(t)p(t)\rangle }\approx \arctan\frac{u_{y}}{u_{x}}$$

where 〈 〉 stands for time averaging, and the approximate expression was obtained using Equation (5). The full 360° azimuth is resolved, taking into account the sign of the numerator and denominator of Equation (6).

If the following assumptions hold: (1) the source is in the far field; (2) 3D propagation effects can be neglected; (3) the frequency of the signal is high compared with the cutoff frequency of the acoustic channel—therefore it acts as a waveguide—and (4) the receiver is far from the boundaries—the method above can be used even in a multipath environment [20]. However, due to multipath, a similar approach cannot be, in general, used to estimate elevation, since the energy generated by the source impinges on the vector sensor in multiple arrivals (echoes) from different elevation angles; thus, the elevation seen by the vector sensor is in some sense only a “mean” elevation [20].

### 2.3. Amplitude-Delay-Angle Estimation in a Multipath Environment

In an underwater environment, it is a common assumption to consider that the multipath structure received on a sensor well away from the channel boundaries is a sum of plane waves. Thus, the ocean acts as a linear system, and neglecting the Doppler, the waveform sampled by the pressure sensor is:

(7) $$p(t)=\sum_{m=1}^{M}a_{m}s(t-\tau_{m})+n(t)$$

where *M* is the number of echoes, *a_m_* and *τ_m_* are, respectively, the strength (amplitude) and time delay of the *m*-th echo and *n*(*t*) represents the additive noise. In the far-field, the pressure and the particle velocity components are in phase [32]; therefore model Equation (7) can be extended to the particle velocity field by:

(8) $$\begin{array}{ccccc} \left[\begin{array}{c} v_{x}(t)\\ v_{y}(t)\\ v_{z}(t) \end{array}\right] & = & \left[\begin{array}{c} \sum_{m=1}^{M}a_{m}^{x}\;s(t-\tau_{m})\\ \sum_{m=1}^{M}a_{m}^{y}\;s(t-\tau_{m})\\ \sum_{m=1}^{M}a_{m}^{z}\;s(t-\tau_{m}) \end{array}\right] & + & \left[\begin{array}{c} n_{x}(t)\\ n_{y}(t)\\ n_{z}(t) \end{array}\right] \end{array}$$

where the coefficients, $a_{m}^{x}$, $a_{m}^{y}$, $a_{m}^{z}$, are the attenuation along the *m*-th path for the three components of the particle velocity. The noise sequences, *n_x_*(*t*), *n_y_*(*t*), *n_z_*(*t*), are additive zero mean, mutually uncorrelated and uncorrelated with the signal, which is a fair assumption when the sensor self-noise is the most relevant noise component. Making the further assumption that the signal, *s*(*t*), is known and has a narrow autocorrelation, a least-squares or maximum likelihood approach for time delay and amplitude estimation can be applied [26]. Given the estimates of $a_{m}^{x}$, $a_{m}^{y}$, $a_{m}^{z}$, the elevation (and azimuth) of the different echoes can be obtained by simple relations.

Considering a snapshot of *N* samples acquired at a sampling interval, Δ*t*, System Equation (8) can be written as:

(9) Y=S(τ)A+N

where **Y** is a matrix of dimension, *N* × 3, whose columns, **v***_x_*, **v***_y_*, **v***_z_*, represent the components of the vector sensor (**Y** = [**v***_x_*|**v***_y_*|**v***_z_*]), amplitude matrix, **A**, is of dimension, *M* × 3, **A** = [**a***_x_*|**a***_y_*|**a***_z_*], **a***_x_*, **a***_y_*, **a***_z_* are the vectors of amplitudes of individual components and matrix **S**(*τ*) has dimension *N* × *M* (*τ* = [*τ*_1_, …*τ_m_*, …, *τ_M_*]), where the *m*-th column is given by **s**(*τ_m_*) = [*s*(−*τ_m_*), …, *s*((*N* − 1)Δ*t* − *τ_m_*)]*^T^*. Matrix **N** of dimension (*N* × 3) represents the noise components (**N** = [**n***_x_*|**n***_y_*|**n***_z_*]).

If the amplitude matrix, **A**, is deterministic, a least squares approach can be used to estimate the amplitudes and time delays [26]. Assuming that the delays are known, the amplitudes are estimated by minimizing the functional:

(10) $$\hat{\text{A}}(\tau )=\arg\{\underset{\text{A}}{\min}{\left\|Y-\text{S}(\tau )\text{A}\right\|}^{2}\}$$

whose solution is given by:

(11) $$\hat{\text{A}}(\tau )={\left[{\text{S}}^{\text{H}}(\tau )\text{S}(\tau )\right]}^{-1}{\text{S}}^{\text{H}}(\tau )\text{Y}$$

where the superscript **H** represents complex conjugate transpose. Since the time delays are generally unknown, the amplitudes are estimated for each plausible time delay, giving rise to a delay-amplitude curve. When the autocorrelation function of the source signal is sharp and the relative time delays are of smaller order than the observation time (*N*Δ*t*), then the envelope of the absolute value of a delay-amplitude curve is known as the arrival pattern. The amplitude-delay estimates of the echoes are given by their *M* highest peaks (absolute value). When the noise is white and the different echoes suffer uncorrelated perturbations, the amplitude-delay estimation procedure can be equivalently obtained by a matched filter [26]; thus, independently for each vector sensor component. In the case of a stationary environment and when several transmissions are available, the amplitude estimates can be obtained by averaging.

Once the coefficients, ${\hat{a}}_{m}^{x}$, ${\hat{a}}_{m}^{y}$, ${\hat{a}}_{m}^{z}$, of the *m*-th echo are estimated, then the corresponding azimuth, *θ̂_m_*, and elevation, *ϕ ^_m_*, are given by:

(12) $${\hat{\theta }}_{m}=\arctan\frac{{\hat{a}}_{m}^{y}}{{\hat{a}}_{m}^{x}}$$

(13) $${\hat{\phi }}_{m}=\arctan\frac{{\hat{a}}_{m}^{z}}{\sqrt{{\left({\hat{a}}_{m}^{x}\right)}^{2}+{\left({\hat{a}}_{m}^{y}\right)}^{2}}}.$$

The elevation estimates, along with the relative echo arrival times, form the basis for the source range-depth estimation algorithm.

### 2.4. Range-Depth Estimation by Backpropagation

The source range and depth backpropagation estimation procedure used in this work was introduced by Voltz and Lu [21] for a vertical hydrophone array. Let us assume an ideal noise-free scenario, where a source is transmitting a signal, and one is able to accurately determine the elevation and associated arrival times of the signal echoes impinging on a receiver. By the reciprocity principle, a ray launched from the receiver at a given angle has the same trajectory as an echo received at that elevation angle. One says that such a ray is backpropagated. One should note that backpropagation, like other model-based methods, requires *a priori* knowledge of the environment (e.g., sound speed profile, bathymetry and bottom parameters) [33].

Source localization is possible by tracing the trajectories of, at least, two echoes impinging on the receiver from different elevation angles and searching for range-depth points, where trajectories intersect each other. Several intersection points can occur along the trajectories; however, the source position can be uniquely determined by using the knowledge of relative time delays between echoes. This can be done by time aligning the different rays, *i.e.*, delaying rays by the estimated relative delays. Since the ray trajectories depend on the sound speed profile and bathymetry, the method is sensitive to uncertainties in those parameters. In practice, estimates of the elevation and travel time are also affected by noise. The estimate, *r̂*, of the source range, *r*, and *ẑ* of the depth, *z*, can be obtained by joint minimization of the mean square error:

(14) $$\hat{r}=\arg\{\underset{r,\tau_{a}}{\min}\sum_{m=1}^{M}{\left[r_{m}(\tau_{a})-r\right]}^{2}\}$$

(15) $$\hat{z}=\arg\{\underset{z,\tau_{a}}{\min}\sum_{m=1}^{M}{\left[z_{m}(\tau_{a})-z\right]}^{2}\}$$

where *τ_a_* is the aligned time, *r_m_*(*τ_a_*) and *z_m_*(*τ_a_*) are, respectively, the range and depth of the *m*-th (*m* = 1 ⋯ *M*) backpropagated ray trajectories at time, *τ_a_*. The well-known solution for this optimization problem is the average range and depth given by:

(16) $$\hat{r}(\tau_{a})=\frac{1}{M}\sum_{m=1}^{M}r_{m}(\tau_{a})$$

(17) $$\hat{z}(\tau_{a})=\frac{1}{M}\sum_{m=1}^{M}z_{m}(\tau_{a})$$

at a time, *τ_a_*, where the range variance, $\sigma_{r}^{2}(\tau_{a})$, and the depth variance, $\sigma_{z}^{2}(\tau_{a})$, are obtained when:

(18) $$\sigma_{r}^{2}(\tau_{a})=\frac{1}{M}\sum_{m=1}^{M}{\left[r_{m}(\tau_{a})-\hat{r}(\tau_{a})\right]}^{2}$$

(19) $$\sigma_{z}^{2}(\tau_{a})=\frac{1}{M}\sum_{m=1}^{M}{\left[z_{m}(\tau_{a})-\hat{z}(\tau_{a})\right]}^{2}$$

jointly attain the minimum. Thus, a possible objective function to be minimized is the sum of variances, *i.e.*:

(20) $$\sigma^{2}(\tau_{a})=\sigma_{r}^{2}(\tau_{a})+\sigma_{z}^{2}(\tau_{a})$$

or, equivalently, its square root, *σ* (aka standard deviation).

This method is numerically very efficient, since it only requires the computation of the trajectory and respective travel time of few rays and a one-dimensional search.

### 2.5. Range-Depth Estimation by the Image Method

**Figure 1.**
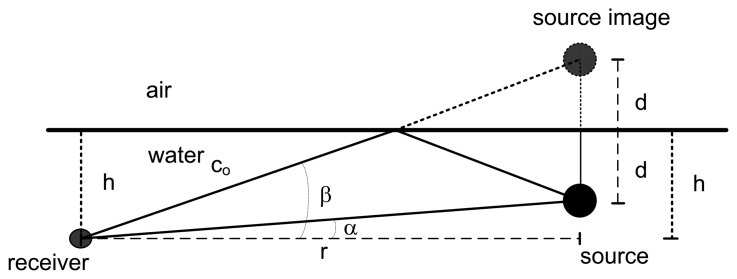
Geometry of the source image method.

Assuming that the sound speed profile is (approximately) isovelocity and that the geometry of the experiment allows for a direct and a surface-reflected echo between the source and the receiver, the source range and depth can be estimated by simple geometric relations based on the source image method (Figure 1). *α* being the elevation of the direct echo and *β* the elevation of the surface-reflected echo as seen by the receiver, the source range, *r*, and depth, *d*, are related by the following equations:

(21) $$\begin{array}{c} r\tan\alpha +d=h\\ r\tan\beta -d=h \end{array}$$

where *h* is the receiver depth. The solution of Equation (21) for *r* and *d* is straightforward. Although limited to a particular geometry, the source image method allows one to determine the range and depth of a source without the need for a ray tracing code, which can be advantageous to implement in light systems.

## 3. Numerical Examples

**Figure 2.**
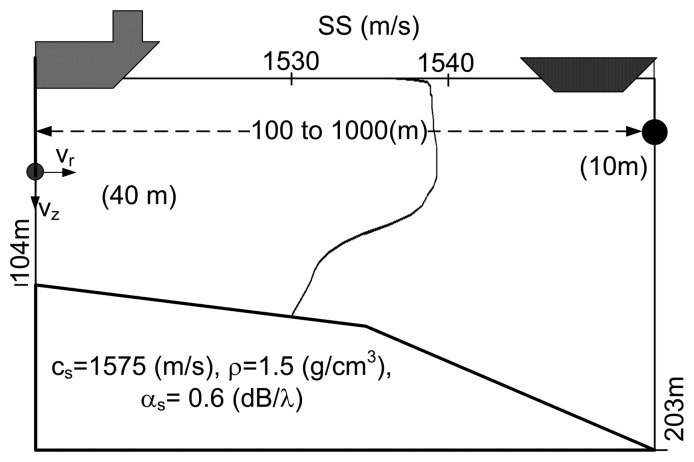
Makai'05 scenario used for the simulation: the source deployed at 10 m depth moves between a 100 and 1,000 m range from the vector sensor deployed at 40 m. The sound speed profile shows a large mixed layer, characteristic of Hawaii, USA. The bottom parameters are those estimated in [10].

**Figure 3.**
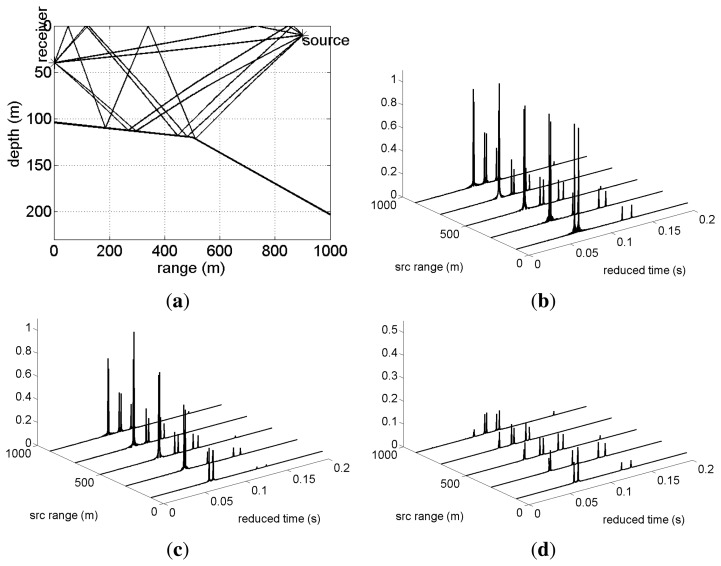
Makai'05 simulation scenario of Figure 2: eigenrays for a source at 900 m (**a**); and arrival patterns for various source ranges—pressure (**b**); horizontal particle velocity (**c**); and vertical particle velocity (**d**).

For testing the methods presented in the previous section and anticipating their performance on real data, the environmental and geometry scenario used for simulation is based on that of the Makai Experiment [34]. The simulation scenario is shown in Figure 2, where a vector sensor is suspended from a research vessel at 40 m depth. The sound source was suspended at 10 m depth and moved along a straight line between 100 m and 1,000 m range from the receiver, transmitting a linear frequency-modulated (LFM) chirp with a duration of 50 ms and a frequency band of 8–14 kHz. The bathymetry is range-dependent with water depth 104 m at the receiver and 203 m at the source when at 1,000 m range. The sound speed profile at the vector sensor location is represented in Figure 2, showing a thick mixed layer with a thermocline starting at 60 m depth. The bottom was modeled as a half-space characterized by the values estimated in [10]: a bottom compressional speed of 1,575 m/s, a density of 1.5 g/cm^3^ and a compressional attenuation of 0.6 dB/λ. In these simulations, it will be assumed that the azimuth is known; thus, only horizontal and vertical particle velocity components are considered. The channel pressure and particle velocity field frequency response were modeled by the cTraceo ray tracing model [35]. The time domain received waveforms were computed by Fourier synthesis. Figure 3 shows the eigenrays (paths of the echoes that impinge on the receiver) when the source is at a range of 900 m (Figure 3(a)) and the arrival patterns for the pressure, horizontal and vertical particle velocity (Figure 3(b–d), respectively). The arrival patterns for the pressure are normalized by the overall maximum, whereas the arrival patterns for the particle velocity components are normalized by the joint overall maximum. Note that the scale used for the particle velocity arrival patterns are different. In the eigenrays plot, one can notice a direct echo and a surface reflected echo, which correspond to the earliest peaks in the arrival patterns plots. These echoes have low angles relative to the horizontal plane containing the source, which decrease with an increasing source-receiver range. This behavior is observed in the particle velocity components, especially in the vertical component, where the amplitude of the peaks in the first cluster decreases as the source gets further way from the receiver. The latter echoes are bottom reflected. These echoes are also clustered in groups of two echoes depending on the number of surface reflections. Bottom reflected echoes have wider angles and lower amplitudes (especially pressure), mainly due to the attenuation in the bottom. Note that in the vertical particle velocity arrival patterns, the latter peaks have relatively higher amplitudes, since for wider angles, the module of sin(Φ) tends to unity. The amplitudes of the different echoes as seen by the vector sensor components illustrate, in the time domain, the spatial filtering capabilities of a single vector sensor.

A number of 100 realizations were generated according to model Equation (8), but limited to a horizontal and a vertical vector sensor component and source ranges from 100 to 900 m. The additive noise was independent for each component and obeyed a Gaussian distribution. Two different signal to noise ratios (SNR), 5 and 20 dB, at the receiver were generated. Since, in the considered geometry, the received energy in the horizontal component is higher than in the vertical component, as can be seen from the arrival structure shown in Figure 3, the SNR is related to the horizontal component, thus representing the worst case scenario. The elevation angles of the four earlier echoes impinging on the vector sensor were estimated independently for the different realizations. Then, the mean and the standard deviation were computed for each echo from the realization ensemble. In order to reduce time and amplitude discretization-related errors, the sampling frequency of the received waveforms (48 kHz) was increased (interpolated) by a factor of 10. The results are summarized in Table 1, where the lines labeled *true* show the values predicted by the forward ray tracing model.

**Figure 4.**
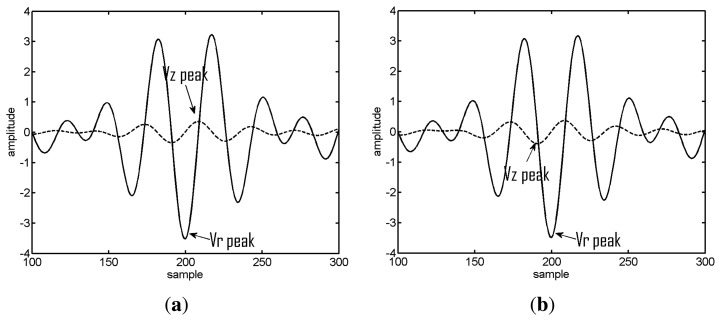
Zoom of the amplitude-delay curve in the neighborhood of the first echo for two signal realizations (5 dB SNR) at a source distance of 300 m showing the expected behavior (**a**) and a sign error (**b**). The horizontal particle velocity (Vr) is represented by the solid line and the vertical particle velocity (Vz) by the dashed line. The arrows indicate the peaks.

The values in brackets represent the standard deviation. The star mark appears when a sign error occurs at least once in the ensemble of realizations for the given echo. It can be seen that the absolute errors are always smaller then 1.2 degrees, and as expected, the standard deviation increases with decreasing SNR. Generally, the SNR at the receiver for distant signals decreases, which, in turn, also contributes to a higher variance of the estimates. Unsurprisingly, the sign error of the estimates increases significantly with decreasing SNR. The autocorrelation function of an LFM chirp is an oscillatory function; thus, due to the noise, the location of the absolute maximum that determines the sign of the elevation estimates can be shifted by an oscillation period, therefore resulting in a sign error. This situation is illustrated in Figure 4, showing the amplitude-delay curve (horizontal and vertical particle velocity) in the neighborhood of the first echo for two realizations at a source range of 300 m. One can observe that the sign of the peak of the vertical particle velocity changed among realizations. This sign ambiguity of the elevation estimates could be, in principle, minimized using a second vector sensor.

**Figure 5.**
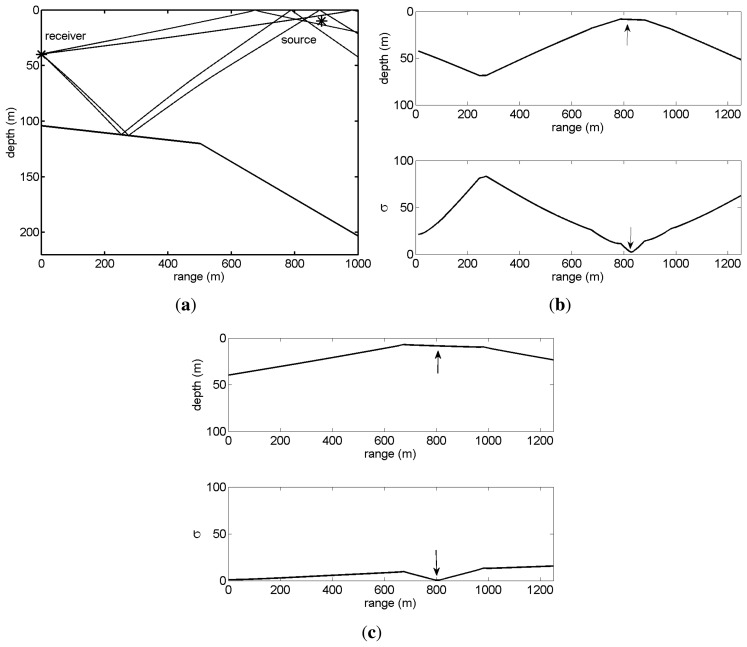
Source localization results for the scenario of Figure 2 using the backpropagation method for source range and depth, 900m and 10 m, respectively and SNR = 5 dB: true source and receiver position (represented by the star) and backpropagated rays (**a**), ambiguity curves (*σ*) and source-localization plot considering four rays (**b**) and two rays (**c**). The arrows in plots (**b**) and (**c**) indicate the estimated source position (upper plots) and the corresponding minimum of the ambiguity curve (lower plots).

Next, using the elevation angle estimates for the 5 dB SNR presented in Table 1 and respective arrival times (not shown), the source range and depth were estimated applying the ray backpropagation and source-image method. For the ray backpropagation method, the estimates were obtained considering three different sets of echoes (the results are summarized in Table 2): all four echoes, the direct and the surface reflected echoes only (column labeled *echoes 1&2*) and the bottom reflected echoes only (column labeled echoes *3& 4*). For the image method only, the direct and surface-reflected echoes are considered. Figure 5 illustrates the backpropagation method when the source is at a 900 m range. Figure 5(a) presents the backpropagated rays, where one can notice that the rays do not intercept at a single point, due to angle and travel time estimation errors. The objective function dependence in the range is plotted for four echoes in Figure 5(b) and for two echoes in Figure 5(c). For each case, the estimated source range is given by the minimum of the objective function and corresponding depth (respectively, lower and upper plots of Figure 5(b,c)). Whereas the objective function for four backpropagated rays has a single sharp minimum (Figure 5(b)) when only two rays are backpropagated, the shape of the objective function is smooth, giving rise to an increased ambiguity (Figure 5(c)), especially in ranges close to the receiver, because of the very short relative time delay between echoes and the close shooting angles.

Generally, range and depth estimation errors increase with source range, since small angle perturbations from the nominal value give rise to larger range depth perturbations. However, the errors are less than 2 m in depth and 75 m in range at the maximum range of 900 m, the worst case considered. The results obtained from the latter echoes, which are bottom reflected, present higher estimation errors than those obtained from direct and surface reflected echoes. Bottom reflection is frequency dependent, which is accounted for by the forward propagation model used to synthesize the received signal; however, the backpropagation uses only the echo path and travel time computed at a given frequency (in general, the middle frequency of the signal band). Moreover, bottom reflected echoes are more attenuated (depending on the bottom structure and the angle of incidence); thus, they are more affected by noise. One can also notice that the source image method gives reasonable estimates in the considered scenario, even at longer ranges, because the sound speed profile is almost isovelocity in the source-receiver layer.

## 4. Experimental Data Results

### 4.1. Experimental Setup

**Figure 6.**
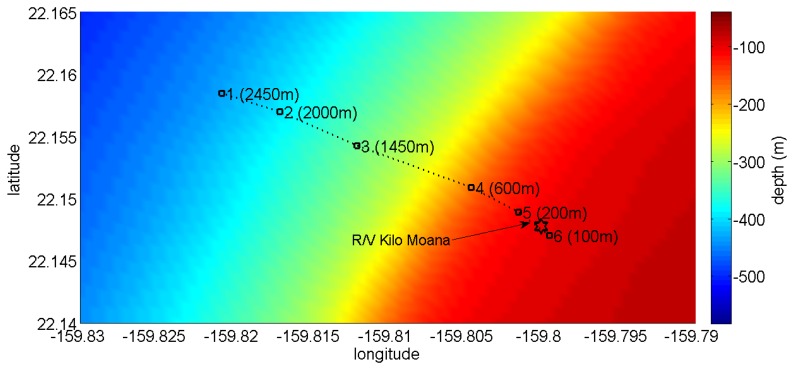
Bathymetry of the Makai'05 experimental area for the field calibration event with the superimposed research vessel R/V Kilo Moana location (pentagon) and GPS fixes of the source rubber boat (square). The values in brackets represent the distance to the R/V Kilo Moana.

**Figure 7.**
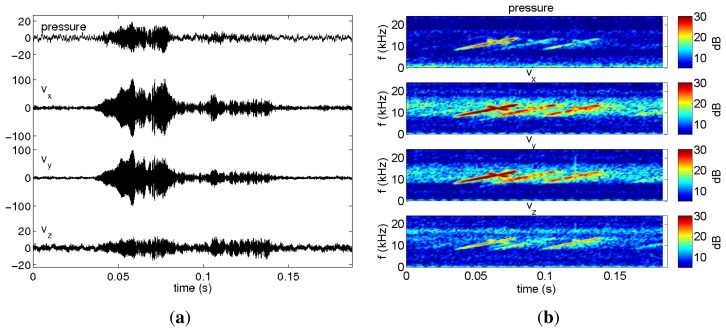
Waveforms received from a 350 m distant source for the various vector sensor components (**a**) and respective spectrograms (**b**).

The data set analyzed here was acquired during the Makai Experiment (Makai'05), which took place off the west coast of Kauai I. in September 2005. The Makai'05 experiment was devoted to high frequency acoustics, and details of the experimental setup are described in [34]. This paper is concerned with the data acquired during the field calibration event, whose geometry is identical to that used in the numerical example (Figure 2). The vector sensor acquisition system used in the experiment was composed by four Wilcoxon TV-001 vector sensors [1,6,22], configured in a vertical array with 10 cm element spacing. The system was suspended off the stern of the research vessel, Kilo Moana, with a 150 kg weight at the bottom, to ensure that the array stayed as close to the vertical as possible. The *z*-axis was vertically oriented downwards, with the deeper sensor at 40 m. In the present data analysis, only the vector sensor at 40 m is considered. In the field calibration event, a Lubell 916C sound source deployed at 10 m depth from a small rubber boat was towed during a period of one hour from a 2.5 km distant point towards the research vessel that was holding a fixed position. Figure 6 shows the rubber boat GPS fixes and the research vessel (R/V) Kilo Moana location superimposed on the bathymetry of the area. The rubber boat track starts at a deeper location, moving along the continental steep slope towards a smooth and uniform area with a water depth of approximately 104 m. The source signals used for localization in this work were transmitted at various locations between approximately 600 m northwest and 100 m southeast of the R/V Kilo Moana, respectively, GPS fixes 4 and 6 in Figure 6. Ground truth measurements were carried out in this area during previous experiments and showed that most of the bottom in the area is covered with coral sands over a basalt hard bottom. The acoustic parameters of the sediment were estimated by Santos *et al.* [10], for the bottom compressional speed, attenuation and density—values already used in Section 3 of this paper. The Lubell 916C sound source transmitted 50 ms long LFM chirps spanning the 8–14 kHz band, transmitted in blocks of 30 chirps from various ranges along the track. The signals were acquired at a sampling frequency of 48 kHz. Due to a technical problem, the time stamp in the data is not synchronized with GPS; therefore, the precise position of the source for the various blocks of acquired data is not available. Figure 7 shows the time series (Figure 7(a)) and spectrograms (Figure 7(b)) of the received waveforms, when the source was at approximately 350 m range, where a strong multipath effect can be seen. The response of the vertical component should also be noted, which emphasizes the latter arrivals when compared with pressure or horizontal components: the relative amplitude of the latter arrival appearing approximately at 0.1 s is in the vertical component, higher than in the other components. This differentiating spatial response of the vertical component was explored in [10,36] to enhance the resolution of bottom parameter estimates. Technical problems with the gain of the pre-amplifiers explain why the amplitude of signals received from larger distances and those that suffer bottom reflections, which are more attenuated, is very low. In the next section, only the transmissions at a source-receiver range smaller than 500 m and the direct and surface reflected arrivals are considered.

### 4.2. Azimuth, Elevation and Travel Time Estimates

The received signal was filtered for a ship noise band (90–350 Hz) and an acoustic source band (8–14 kHz) by linear phase bandpass filtering. The ship noise was used to determine the orientation of the *x*-axis relative to the ship. For this purpose, the azimuth of the ship noise was estimated using the intensity-based method. The estimates were obtained by applying Equation (6) to ship noise received simultaneously with LFM chirps. The azimuth estimates are presented in a reference frame, where the *x*-axis is aligned west-east and the *y*-axis is aligned south-north. The elevation angles are considered positive towards the surface. The azimuth estimates obtained from the ship noise are very stable along the whole experiment [24,37].

**Figure 8.**
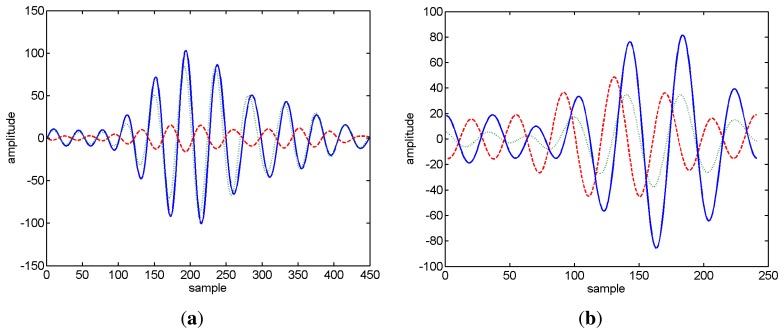
Zoom of the amplitude-delay curve in the neighborhood of the first echo for the *x*-component (solid line), *y*-component (dotted line) and *z*-component (dashed line) at approximate source-receiver distances of 250 m (**a**) and 350 (**b**).

As a first step to localize the source, the arrival times and amplitudes of the various echoes impinging on the vector sensor from each transmitted chirp were estimated from the arrival patterns using Equation (11). Since the transfer function of the transmission chain was unknown, a simulated undistorted LFM chirp was used in the amplitude arrival time estimation procedure. In order to increase the travel time and amplitude resolution, the acquired signal was up sampled by a factor of 10, becoming the virtual sampling frequency of 480 kHz. Figure 8 shows the delay-amplitude curve in the neighborhood of the first echo for the three vector sensor components at two different source ranges, illustrating that the travel time of an echo varies significantly among vector sensor components. While in Figure 8(a), the various components are nearly in phase, in Figure 8(b), a significant deviation occurs. Data inspection revealed that the perturbations varied among distances and among echoes, but were stable for the same echo number among transmissions at a given distance. Thus, for azimuth and elevation estimates, using Equations (12) and (13), respectively, the different components were treated independently. The amplitude of a given component was considered, where its absolute maximum occurs, whereas the associated travel time to be used in the backpropagation algorithm was that of the *z*-component. For the reasons explained above, only the direct and surface-reflected echoes were used for source range-depth estimation. For each distance, six chirps in the signal block were processed; azimuth and elevation were estimated. For the azimuth estimation only, the direct echo was considered. Table 3 presents the mean values and the standard deviations of those estimates, and for the sake of clarity, the range estimates are discussed in next section. As discussed in the numerical examples, the sign of the estimates suffer from a large ambiguity; thus, the sign of the elevation angle of the chirp echoes was assigned using the previous knowledge of the geometry. However, the sign of the azimuth of the ship noise was determined directly from the data. The orientation of the vector sensor relative to the R/V Kilo Moana given by the azimuth estimates from the ship noise was stable along time. This behavior was also observed for the other Makai'05 events for a full vector sensor array [37] and a single vector sensor [24]. One should note that the standard deviations of the elevation angles are lower for direct than for surface reflected echoes, which have a smaller amplitude and are subject to perturbations induced by the ocean surface. The high standard deviation of azimuth estimates at position min 54 and min 57 can be explained by the shorter range. A horizontal displacement at a shorter range gives rise to a higher azimuth perturbation when compared with similar displacement at a longer range.

### 4.3. Range-Depth Estimates

**Figure 9.**
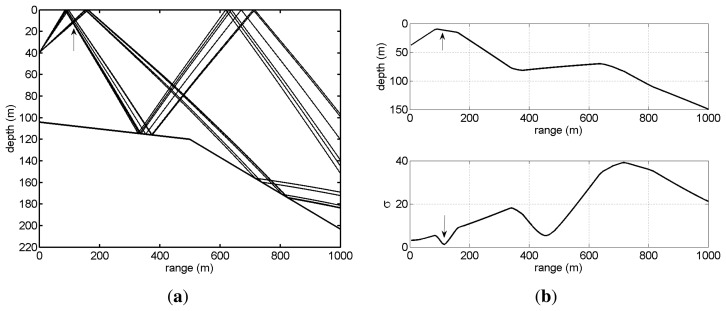
Makai'05 source range-depth estimation at min 57 of the field calibration event considering six sets of backpropagated rays (12 rays) (**a**). The ambiguity curve (**b**) has the minimum (indicated by the arrow) at the 114 m range, lower plot, corresponding to 11.5 m depth in the upper plot.

The range and depth of the source were estimated with the ray backpropagation method using the elevation angles of the direct and surface reflected echoes and respective relative arrival times. In order to obtain an estimate of the standard deviation of the estimates, the objective function is an average of the objective functions computed for each single realization of the transmitted chirp signal. Figure 9(a) shows the direct and surface reflected backpropagated rays with elevation angles estimated from six chirps transmitted at min 57, where the source was at a range of 114 m. The overall objective function (ambiguity curve) dependence in the range computed from these rays (and travel time estimates) is shown in the lower plot of Figure 9(b), whereas the estimated source range is given by the minimum of the objective function and the corresponding depth in the upper plot. The range and depth estimates at various source distances obtained by backpropagation are presented in Table 4. Column *σ* represents the minimum of the square root of the objective function, where the smaller values (smaller variance), are attained at closer ranges. Model-based source localization methods are, in general, not considered for real-time implementations, because of the time needed to compute the optimization procedure, which requires a large number of forward model runs, but a non-optimized Matlab implementation of the ray backpropagation method took less than 4 s in a current laptop. It is expected that further code optimization would allow for real-time application.

**Figure 10.**
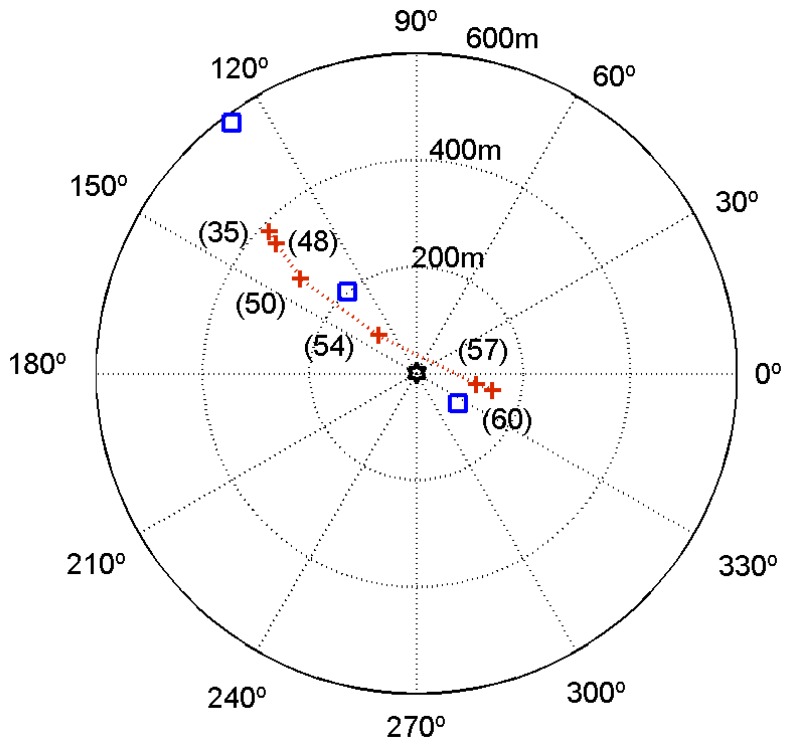
Estimated source location (cross marks) relative to the R/V Kilo Moana (located at the origin) of the Makai'05 field calibration event. The square marks indicate GPS fixes obtained with a handheld GPS on board the source rubber boat. The values in brackets represent the time (in min) of Tables 3 and 4.

For comparison purposes the results obtained using the source image method are also shown in Table 4. At longer distances, the difference between the estimates obtained by both methods increases, due to the cumulative effect of considering a constant sound speed with the image method. One should remark that the source depth estimates are in close agreement with the nominal depth of the source of 10 m. Figure 10 shows a polar plot with the location of the source using the source range and azimuth estimates, which are represented by stars. The squares represent the positioning of the source relative to the R/V Kilo Moana (at the origin) estimated from the ship's GPS and a handheld GPS device on the source's boat. Unfortunately, the handheld GPS device had no recording capabilities; thus, the source path between GPS fixes is uncertain. Since the range estimates by GPS and by acoustics are affected by some offset, because of the different location of the vector sensor and GPS on board the R/V Kilo Moana, and there is no time stamp in the acoustic data, this did not allow for synchronization between acoustic and GPS data. The source track derived from acoustic data are in relatively close agreement with GPS fixes.

## 5. Conclusions

This paper illustrates the spatial filtering capabilities of a vector sensor applied to source localization of a known broadband signal in a multipath environment. It was shown that the estimation of the angle of arrival (elevation) of a single echo was possible. Given the estimates of the amplitudes of the echoes in the *v_x_*,*v_y_* and *v_z_* vector sensor components, a method to estimate the source azimuth and the elevation was presented. The elevation angles of the direct and surface reflected echoes were used to estimate source range and depth localization by a ray backpropagation algorithm. The method was discussed in the context of simulated data and for a real data set acquired during the Makai Experiment. It was shown that for ranges below 500 m, it was possible to estimate the source range and depth in agreement with the known geometry of the experiment. To the best of our knowledge, this is the first work in open literature that reports 3D localization results with a single vector sensor in a shallow water environment. In comparison with other model-based methods discussed in the literature for source localization using a single device (hydrophone), the present method explores the spatial filtering capabilities of a single vector sensor to significantly reduce the number of forward model runs; thus, it can be potentially implemented in low end or light real-time systems.

## Figures and Tables

**Table 1.** Estimated angles of the four earliest echoes impinging on the vector sensor at different source distances as given by the forward model (true) and estimated considering an signal to noise ratios (SNR) of 20 and 5 dB. The values in curved brackets represent the standard deviations. The star mark indicates that at least one sign error occurred in the ensemble of estimates for the given echo.

**Source Depth 10 m**		**Echo Number**				
**Range [m]**	**SNR [dB]**	**1 [°]**	**2 [°]**	**3 [°]**	**4 [°]**	
100	True	16.3	26.1	−60	−63	model
	20	17.3 (0.1)	27.3 (0.1)	−59.4 (0.5)	−62.5 (0.7)	estimate
	5	17.3 (0.4)	27.4 (0.4)	−59.4 (3.4) *	−62.1 (3.7) *	estimate

300	True	5.6	9.3	−30.8	−33.8	model
	20	5.9 (0.1)	9.9 (0.1)	−31.8 (0.4)	−34.4 (0.3)	estimate
	5	5.9 (0.4) *	10.1 (0.5)	−31.9 (2.3) *	−34.5 (2.0) *	estimate

500	True	3.3	5.5	−20.7	−22.8	model
	20	3.7 (0.1)	5.8 (0.1)	−21.8 (0.3)	−23.8 (0.3)	estimate
	5	3.7 (0.5) *	5.8 (0.5)	−21.9 (1.5) *	−24.0 (2.1) *	estimate

700	True	2.3	3.9	−15.9	−17.6	model
	20	2.5 (0.1)	4.1 (0.1)	−16.8 (0.2)	−18.8 (0.4)	estimate
	5	2.7 (0.6) *	4.1 (0.4) *	−16.8 (1.3) *	−18.7 (2.0) *	estimate

900	True	1.8	2.9	−13.2	−14.5	model
	20	1.9 (0.1)	3.1 (0.1)	−14.1(0.2)	−15.2 (0.2)	estimate
	5	2.1 (0.5) *	3.2 (0.5) *	−14.1 (1.0) *	−15.3 (1.0) *	estimate

**Table 2.** Estimates of range and depth of a simulated source at 10 m depth, between a 100 and 900 m range from the receiver. The backpropagation method was applied to the four echoes, the earlier two echoes (1& 2) and the last two echoes (1& 2). The source image method was applied to the earlier two echoes. The range and depth estimates were obtained using estimates of the elevation angles from simulated data with 5 dB SNR.

**Simulated**	**Ray Backpropagation**						**Image Method**	
**Depth 10 m**	**4 Echoes**		**Echoes 1&2**		**Echoes 3 &4**		**Echoes 1&2**	

**Range** **[m]**	**Range** **[m]**	**Depth** **[m]**	**Range** **[m]**	**Depth** **[m]**	**Range** **[m]**	**Depth** **[m]**	**Range** **[m]**	**Depth** **[m]**
100	102.5	10.0	99.4	10.2	104.3	9.7	95.7	9.9
300	299.3	10.2	300.1	11.3	290.3	8.9	282.1	10.5
500	499.4	9.8	500.9	9.5	469.1	9.4	477.6	8.8
700	649.1	9.6	668.1	8.2	640.2	8.3	649.5	10.9
900	829.2	8.6	804.5	8.4	830.1	8.4	857.7	8.3

**Table 3.** Mean azimuth and elevation estimates obtained at various instants of the Makai'05 field calibration event for the ship noise (azimuth only) and for the broadband sound source at 10 m depth and a range between 100 and 400 m (estimated from acoustic data, Figure 10). The values in brackets represent the estimated standard deviation.

**Time[min]**	**Source Range[m]**	**Azimuth[°]**		**Elevation[°]**	
		**Ship Noise** **90–350 Hz**	**Source** **8–14 kHz**	**Dir.Echo**	**Surf.Ref.Echo**
35	386.3	132.4	136.1 (0.2)	4.1 (0.1)	7.5 (0.1)
48	357.5	132.2	137.3 (0.8)	4.9 (0.1)	7.5 (0.7)
50	279.8	134.4	140.9 (0.4)	5.5 (0.3)	9.8 (1.3)
54	100.8	133.1	135.1 (2.3)	16.7 (0.2)	25.7 (2.0)
57	114.2	131.7	−10.2 (1.0)	14.0 (0.4)	23.8 (1.0)
60	145.4	132.2	−12.6 (0.6)	12.4 (0.2)	17.8 (1.1)

**Table 4.** Source range and depth estimates at various instants of the Makai'05 field calibration event using the ray backpropagation method and the image method. The column marked, *σ*, represents the minimum of the square root value of the objective function used with the backpropagation method. The true source depth is 10 m. The estimated range values compare with the GPS fixes in Figure 10.

**Time [min]**	**Ray Backpropagation**			**Image Method**	

	**Range [m]**	**Depth [m]**	***σ* [m]**	**Range [m]**	**Depth [m]**
35	386.3	10.54	6.7	391.1	11.7
48	357.5	10.55	9.4	367.1	8.3
50	279.8	11.1	1.8	298.8	11.0
54	100.8	10.1	1.5	101.9	9.2
57	114.2	11.5	1.0	115.0	11.0
60	145.4	7.9	1.2	146.7	7.3
